# General Practice Care for Patients with Rare Diseases in Belgium. A Cross-Sectional Survey

**DOI:** 10.3390/ijerph15061180

**Published:** 2018-06-05

**Authors:** Nicole Boffin, Elfriede Swinnen, Johan Wens, Montse Urbina, Johan Van der Heyden, Viviane Van Casteren

**Affiliations:** 1 SD Epidemiology and Public Health, Sciensano, 1050 Brussels, Belgium; elfriede.swinnen@sciensano.be (E.S.); purbina@sciensano.be (M.U.); Johan.VanderHeyden@sciensano.be (J.V.d.H.); Viviane.vanCasteren@sciensano.be (V.V.C.); 2 Department of Medicine and Health Sciences, Primary and Interdisciplinary Care, University of Antwerp, 2610 Antwerp, Belgium; johan.wens@uantwerpen.be

**Keywords:** family practice, chronic disease, epidemiology, referral and consultation, health surveys, workload

## Abstract

There are almost no studies about rare diseases in general practice. This study examined care characteristics of active rare disease patients in the Belgian Network of Sentinel General Practices (SGP) and the importance of rare diseases in general practice by its caseload, general practitioner (GP)–patient encounter frequency and nationwide prevalence. The SGP reported data about: (i) the number of active rare disease patients in 2015; and (ii) characteristics of one to three most recently seen patients. Rare diseases were matched against Orphanet (www.orpha.net). GP encounter frequency and patients’ age were compared to the total general practice population. Details from 121 active patients (median age: 44, interquartile range (IQR) 24–60) showed that for 36.9% the GP had been the first caregiver for the rare disease and for 35.8% the GP established a diagnostic referral. GPs rated their knowledge about their patients’ disease as moderate and used Orphanet for 14.9% of patients. Any active rare disease patients (median: 1, IQR 0–2) were reported by 66 of 111 SGP. Compared to the total general practice population, the mean GP encounter frequency was higher (7.3; 95% confidence intervals (CI) 6.1–8.5 versus 5.4; 95% CI 5.4–5.4). The prevalence of rare diseases in the Belgian general practice population was estimated at 12.0 (95% CI 10.3–13.9) per 10,000. This study acknowledges the important role of GPs in rare disease care. Knowledge and use of Orphanet by GPs could be improved.

## 1. Introduction

During the past two decades, rare diseases have become a major public health concern, largely as a result of advocacy by patient organizations, physicians, researchers and policy makers. The European Organisation for Rare Diseases (EURORDIS) showed that rare disease patients face many obstacles [1]. It has been argued that general practitioners (GPs) have an important role in alleviating the heavy burden on these patients [2,3]. A symposium on primary care for rare disease patients guided this study [4].

In Europe, rare diseases were defined by the threshold for rarity as “life threatening or chronically debilitating conditions that affect no more than 5 in 10,000 people” [5]. An estimated 6% to 8% of the European population is affected by a rare disease [6]. However, little is known about the magnitude of the rare disease patient population and the care they receive in general practice. A PubMed search (January 2017) resulted in six original research articles on rare diseases in the general practice population (see Figure S1) [7,8,9,10,11,12]. Browsing the citations resulted in one additional Dutch study [13]. Our description of seven cross-sectional studies (see Table S1) shows that only two describe data using the aforementioned EU definition [12,13].

In view of the above, we conducted a retrospective study on rare disease patients in the Belgian Network of Sentinel General Practices (SGP) in 2015. Accordingly, our study objectives were to:

- Examine care characteristics of rare disease patients; and
- Examine the importance of rare diseases in general practice by its caseload, i.e., the number of cases in the SGP network, its estimated prevalence in the Belgian general practice population and by comparing the GP-encounter frequency among rare disease patients in the SGP and the Belgian general practice population. We also compared patients’ age in the two populations.

## 2. Materials and Methods

### 2.1. Settings and Participants

Data were collected by the Belgian network of SGP, developed in 1979 drawing on international experiences of sentinel surveillance [14]. In 2015, the SGP network comprised 129 general practices with 182 GPs who purposively recorded clinical care data for the surveillance of health (care) problems. This type of sentinel networks allows collecting contextual information that is not available from patients’ electronic health records (EHR). The SGP network covered 1.4% of the Belgian population throughout all regions and the sentinel GPs are fairly comparable to other GPs [15]. The Belgian network of SGP was approved in its entirety by the Ethical Committees of the Scientific Society of Flemish GPs and the Catholic University of Louvain (UCL).

### 2.2. Data Collection

In January 2016, we invited the SGP to participate in this ad hoc study. Study details and instructions were described on a structured form followed by the opening question whether there had been any active rare disease patients in the practice in 2015, and, if any, their (approximate) number. Rare diseases were described as “diseases occurring in less than 1 of 2000 persons, mostly genetic in nature, usually severe with a chronic and degenerative course”. Patients were considered to be active if there had been at least one GP–patient encounter in 2015 or a care contact about the patient’s care with health care professionals. Three forms were included in the mail-out to report characteristics of at the most three patients that were seen most recently.

### 2.3. Measurements

#### 2.3.1. The Patient Level

The form started with an open question for the name of the rare disease and continued with information about the patient, i.e., gender and age, number of GP–patient encounters and care contacts in 2015, length of the GP–patient relation (computed from the year of first contact), duration of the disease (symptoms) in years (computed from the year of the first suspicion of rare disease), whether the GP was the first caregiver contacted following the onset of symptoms, and whether the diagnosis was confirmed and , if so, in what year. We defined diagnostic delay as an interval of one year or more between the first suspicion and the diagnostic confirmation. The question whether it was the GP who referred the patient to the facility where the diagnosis was established was followed by three possible reason(s) for a negative answer: diagnosis was already known at the first GP–patient encounter, the patient was referred by the GP to another facility, and the referral was initiated by another care provider or by the patient or his/her family. The SGP were asked to rate their knowledge and the usefulness of Orphanet information about each patient’s rare disease on a Likert scale from 1 (very low) to 5 (very high), preceded by the option “?/NA”, described as ”do not know or unknown (“?”) and “not applicable or not used” (“NA”). Orphanet (http://www.orpha.net) is the reference portal for information on rare diseases.

#### 2.3.2. The Level of the Sentinel General Practices

Characteristics of the SGP included in this study were age and gender of the sentinel GPs, the number of weekly patient encounters and the number of reporting (trainee) GPs in the SGP, all aggregated on the practice/SGP level using median values. The SGP were also described by region (Flanders/Wallonia-Brussels), population density of the SGP municipality (low or mixed/high), practice form (solo/group) and use of certified EHR (yes/no).

### 2.4. Analysis

Co-authors E.S. and M.U., both familiar with rare disease coding, independently matched all rare disease names against the April 2016 version of the Orphanet classification. Cases were excluded if their condition was exceeding a prevalence of 5 per 10,000 persons. An inclusive attitude was adopted in the sense that generic (rare) disease names given by the SGP were no reason for exclusion. Results were compared and, if necessary, discussed until consensus was reached.

Logistic regression was used for determinants of study response. For determinants of the number of rare disease patients per SGP, we report incident rate ratios (IRRs) with 95% confidence intervals (CI). To model the SGP characteristics, we used a Vuong test and a test of dispersion to select the best-fit model (Poisson model, zero-inflated or standard negative binomial model).

The prevalence of rare disease patients in the Belgian general practice population was estimated by dividing the (estimated) number of rare disease patients in the SGP by the sum of person years covered by the SGP in 2015, using a Poisson distribution to calculate 95% CI. For 14 SGP with a missing/unknown number of active rare disease patients, we used the number of patients for which characteristics were reported (maximum three). The caseload in 18 non-responding SGP was imputed using the mean number of rare disease patients by practice organization as 82% of group practices and 48% of solo practices reported one or more active rare disease patients (*p* = 0.001).

We compared the mean number of patient encounters in the study population with the total population having had at least one GP encounter in 2015 under the fee-for-service system using a publicly available random sample of the Belgian Compulsory Health Insurance (BCHI) (http://www.aim-ima.be/Permanente-steekproef-EPS).

## 3. Results

### 3.1. Study Participation of the Sentinel General Practices

**Figure 1:**
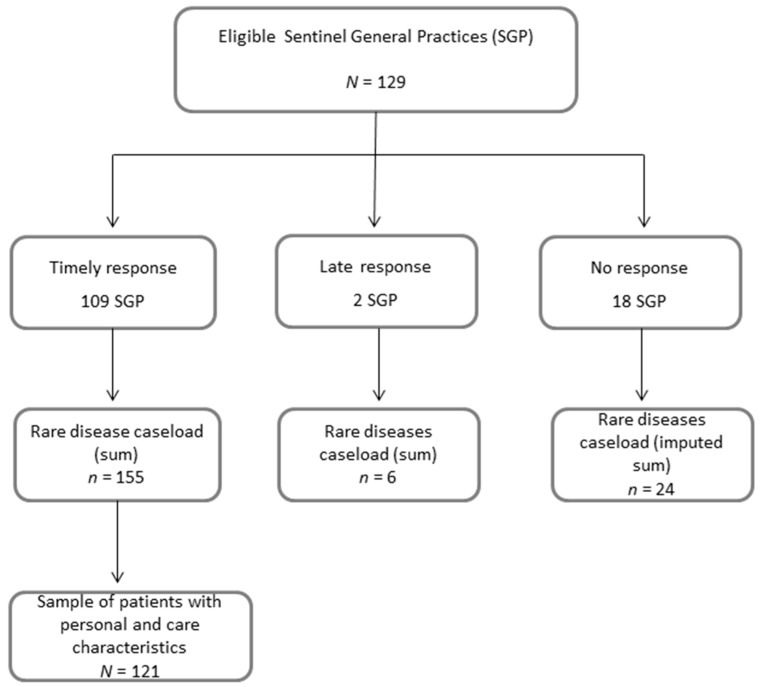
Study dataflow by Sentinel General Practices (SGP) and reported cases.

Response to the survey was obtained from 111 of 129 SGP (86.1%) (Figure 1). From two late responders we only used the (approximate) number of active rare disease patients in 2015. A higher (than median) age of the GPs was the only determinant of study response (Odds Ratio (OR) 6.5; 95% CI 2.23–19.14).

### 3.2. Personal and Care Characteristics of the Sample of Rare Disease Patients

We excluded nine patients not having a rare disease according to Orphanet and three patients that had not been active in 2015. The study thus includes characteristics from 121 rare disease patients reported by 109 SGP (Figure 1). Ninety-nine rare diseases were reported, of which three not fully confirmed due to incomplete information (see Table S2). Characteristics of 121 rare disease patients are described in Table 1.

Median patient age at first contact was 29 (interquartile range (IQR) 6–47). Twenty-one persons (18.8%) were practice patients since their birth. If there was no diagnosis in the year of the first suspicion of a rare disease (24.5%), there was a median diagnostic delay of one year (IQR 1–3).

The GP knowledge about the patients’ rare diseases was moderate (median: 3, IQR 2.5–4). The usefulness of Orphanet was only rated for 14.9% of the patients’ disease. On the SGP level, 14 of 62 SGP rated their knowledge of their patients’ rare diseases as (very) good and 10 of 64 SGP had used Orphanet for information about their patients’ condition.

### 3.3. Caseload by Sentinel General Practices

One or more active rare disease patients were reported by 66 of 111 SGP (59.5%), resulting in a total caseload of 161 rare disease patients. The remark that rare disease patients may have been missed patients was made by 14 SGP, and relatively more by SGP with cases (12 of 14, *p* = 0.032). The median number of rare disease patients per practice was 1 (IQR 0–2). Four of eight SGP characteristics were (borderline) statistically significantly associated with the number of active rare disease patients (Table 2). The use of certified EHR-systems and a group practice organization were independent positive determinants of the SGP caseload.

### 3.4. General Practitioner Encounter Frequency by Rare Disease Patients Compared to the Total General Practice Population

The mean number of patient encounters in 2015 was significantly higher among rare disease patients than in the total general practice population, particularly in the age groups 25 to 64 years (Table 3).

While the encounter frequency increased by age in the total general practice population, it did not among rare disease patients. Table 3 also shows that the population of ≥65 years was relatively much smaller in the rare disease patient population.

### 3.5. Prevalence of Rare Disease Patients in Belgian General Practice

Using the patient population covered by the SGP and the (estimated) number of rare disease patients in the SGP network (including imputed cases), the estimated prevalence of rare disease patients in Belgian general practice was 12.0 (95% CI 10.3–13.9) per 10,000.

## 4. Discussion

We found long-term and often lifelong relations between GPs and their rare disease patients and a considerable role of the GP in the diagnosis of the rare disease. Diagnostic delay was relatively low as 75% of the patients received a diagnosis within a year after first suspicion. The GPs rated their knowledge as (very) good for only 29% of the rare diseases. Orphanet had been used by only 10 of 64 SGP with cases. Active rare disease patients were reported by 60% of the SGP. Compared to the total general practice population, the GP–patient encounter frequency was high, especially among adults between 25 and 64 years. We found that there were much fewer rare disease patients of ≥65 years compared to the total general practice population. The estimated prevalence of active rare disease patients in Belgian general practice (12.0 per 10,000 patients) is much lower than the usual estimate of 6% to 8% affected people in the European population.

This is the first profound study about rare diseases in general practice. Based on characteristics of real-life patients in a longstanding network of experienced SGP, our study method is an alternative to estimate the prevalence of rare diseases. Indeed, examining EHR from GP networks has shown to fail since the International Classification of Primary Care (ICPC) has no codes for rare disease patient care elements [12]. It seems that rare diseases are under-represented in all healthcare coding systems [16].

Our study has several weaknesses. We only verified the rare disease status of a sample of patients. This sample may be biased, e.g., toward patients with frequent contacts. We did not relate our findings to types of rare diseases, e.g., by severity or impact on quality of life. There was no predefined protocol for the inclusion/exclusion of disease names lacking sufficient detail. Only nine false positive cases were excluded, suggesting that GPs are well acquainted with the rare disease concept. However, true-positive rare disease patients may have been missed. In fact, more than 10% of the SGP made remarks about the uncertain number of patients, often mentioning that the reason lies in the absence of diagnostic codes to search EHR for. A prospective study during one year in the complete network may have resulted in a more precise, possibly higher, number of active rare disease patients.

There are several possible explanations for the large difference between our prevalence estimate of 0.1% and the usual estimate of 6% to 8% rare disease patients in the European population [6]. The latter estimate not only includes primary care patients but also patients at all levels of care and non-active patients or relatively healthy persons. Underreporting by the SGP may also explain the gap, together with an overestimation of the European prevalence. To our best knowledge, the latter estimate has never been elucidated with clear evidence. However, our prevalence estimation needs to be confirmed.

There are few studies to which we can compare our findings. We found that previous studies about rare diseases in general practice showed a limited quality of reporting according to STROBE guidelines [17]. The study by van de Laar et al. may be well reported, but we cannot compare findings because the study failed to identify rare disease patients in a sample of EHR [12]. EURORDISCARE 2 (2004) found that 25% of patients with one of eight rare diseases had to wait between 5 and 30 years for a confirmed diagnosis [18]. We found that 25% of the patients had to wait from one to three years. The relatively low rate of diagnostic delay may be typical for rare diseases seen in primary care but diagnostic delay rates may also have decreased over time. The lack of acquaintance with Orphanet is remarkable, certainly since less than one in four SGP rated their knowledge of their patients’ rare disease(s) as (very) good. According to one study we reviewed, a relatively large proportion of rare disease patients felt their GPs were insufficiently qualified to treat them [13]. Another study found that most rare disease patients are realistic about their physician’s knowledge and appreciate honesty about a lack of knowledge [19].

It seems advisable to promote awareness of Orphanet and its use among GPs. In addition to our quantification, the qualification of rare disease problems in general practice merits future research. Thus far, it remains unknown whether GPs experience rare disease patients as burdensome and how experiences are associated with characteristics of patients, rare diseases and involved caregivers.

## 5. Conclusions

To our knowledge, this is the first report on the prevalence of active rare disease patients in general practice and characteristics of a sample of affected patients. The prevalence may be low, but GP involvement is high, as shown by the GP–patient encounter frequency and GPs’ role in their care process.

## Figures and Tables

**Table 1:** Personal and care characteristics of a sample of 121 active rare disease patients in the Belgian Network of Sentinel General Practices (SGP) in 2015.

Variable	Median (IQR)	*n/N* (%)
Patient age (*N* = 121)	44 (24–60)	-
Number of GP–patient encounters in 2015 (*N* = 118)	5 (3–10)	-
Number of care contacts (with other health professionals) about patient in 2015 (*N* = 112)	2 (1–5)	-
Length of patient-GP relation in years (*N* = 112)	14 (5–19)	-
Duration of disease (symptoms) in years (*N* = 97)	8 (3–16)	-
Patient gender: female	-	71/120 (59.2)
GP was first caregiver seen for rare disease (symptoms)	-	41/111 (36.9)
Diagnosis was confirmed	-	116/117 (99.2)
Diagnostic delay (≥1 year between first suspicion and confirmation of diagnosis)	-	23/94 (24.5)
GP referred patient to facility where diagnosis was established	-	43/120 (35.8)
Reasons(s) why GP referral was unnecessary/unsuccessful		
Diagnosis was already known	-	40/77 (52.0)
Referral by another caregiver or self-referral	-	24/77 (31.2)
GP had first referred to other care facility	-	6/77 (7.9)
GP’s medical knowledge of disease is (very) good	-	34/116 (29.3)
Usefulness of Orphanet information about the patient’s rare disease is not applicable/no use	-	103/121 (85.1)

Note: Abbreviations: IQR = Inter Quartile Range. GP: general practitioner (GP).

**Table 2:** Characteristics of Sentinel General Practices associated with caseload of rare disease patients in the Belgian SGP network in 2015 (*N* = 111).

	Rate Ratio for Number of Cases (95% Confidence Intervals (CI))	Adjusted Rate Ratio for Number of Cases (95% CI) ^2,3^
SGP gender composition (*N* = 110)		
<50% men	No significant model obtained	
≥50% men		
SGP age composition (*N* = 110)		
<median	No significant model obtained	
≥median		
Region (*N* = 111)		
Wallonia or Brussels	No significant model obtained	
Flanders		
Population density of SGP municipality (*N* = 111)		
Low or mixed	1.84 (1.12–3.00)	
High	ref	
Use of certified electronic health records (EHR (*N* = 110)		
Yes	4.05 (1.55–10.60)	2.85 (1.09–7.45)
No	ref	Ref
Practice organization (*N* = 111)		
Group practice	2.37 (1.50–3.76)	2.05 (1.29–3.28)
Solo practice	ref	ref
Number of reporting (trainee) GPs (*N* = 110)		
>1	1.76 (0.97–3.18)	
1	ref	
Number of weekly patient contacts in 2015 (*N* = 111)		
≥median	No significant model obtained	
<median		

Note: ^1^ No significant regression models were obtained for independent variables gender and age composition, region, and the number of weekly patient contacts in the SGP as dependent variable; ^2^ SGP characteristics that were significantly (*p* < 0.05) associated at the univariate level were included in the multivariate analysis. The number of reporting (trainee) GPs was significantly associated with the practice organization, therefore it was omitted from multivariate analysis; ^3^ Interaction effects could not be estimated due to the low prevalence of non-use of certified EHR systems.

**Table 3:** Mean number of GP–patient encounters by patient age groups and age distribution in a sample of 208,029 patients from the total general practice population covered by the Belgian Compulsory Health Insurance (BCHI) and a sample of 121 active rare disease patients in the Belgian SGP network in 2015.

		General Practice Population (BCHI)	SGP Rare Disease Population
		*N* = 208,029	*N* = 121 ^1^
Age groups (4)		Mean number of GP encounters (95% CI) ^2^	
	≤24	3.3 (3.3–3.4)	6.0 (2.6–9.4)
	25–44	**4.1 (4.1–4.2)**	**8.4 (6.0–10.8)**
	45–64	**5.3 (5.3–5.3)**	**7.3 (5.6–9.0)**
	≥65	8.9 (8.8–8.9)	8.2 (5.9–10.5)
	Total	**5.4 (5.4–5.4)**	**7.3 (6.1–8.5)**
Age groups (4)		Age distribution [Column % (95% CI)] ^2^	
	≤24	23.8 (23.6–24.0)	28.1 (20.3–37.0)
	25–44	24.1 (23.9–24.3)	23.1 (16.0–31.7)
	45–64	28.5 (28.3–28.7)	35.5 (27.0–44.8)
	≥65	**23.6 (23.4–23.7)**	**13.2 (7.8–20.6)**
	Total	100%	100%

Note: ^1^ The number of GP encounters was missing from 3 of 121 patients. ^2^ Non-overlapping 95% Confidence Intervals (CI) are in bold.

## Supplementary Materials

The following are available online at http://www.mdpi.com/1660-4601/15/6/1180/s1, Figure S1: Flow chart of search for original studies on rare diseases in general practice, Table S1: Characteristics of seven original studies about rare diseases in general practice identified by a literature search (described in Figure S1), Table S2: List of 99 rare diseases reported for 121 active patients in the Belgian Network of SGP in 2015.
