# Supplementary material for: General Practice Care for Patients with Rare Diseases in Belgium. A Cross-Sectional Survey
Source: Int J Environ Res Public Health. 2018 Jun 5;15(6):1180. doi: 10.3390/ijerph15061180 (PMC6025074; doi:10.3390/ijerph15061180)
Supplement: Supplementary file 1 [file ijerph-15-01180-s001.pdf]

International Journal of
Environmental Research and Public Health

MDPI

Table S1. Characteristics of seven original studies about rare diseases in general practice identified by a literature search (described in Figure S1)

Reference	Place, years	Population sample	Study aims/objectives	Methods	Use of European rare disease definition	Results about caseload, number of cases or prevalence
Avellaneda Fernández *et al*. [7]	Spain, Madrid, 2004	8 groups of GPs from an urban area and 1 rural based GP	Describe the need for rare disease training in PC	In-depth interviews and group dynamics	EU definition was one of the subjects that was explored	No data
Avellaneda Fernández *et al*. [8]	Spain, 2008-10	260 (of unknown total) Primary Health Care Physicians (PHCP)	Describe perceptions of PHCP regarding rare diseases, and the characteristics of these patients	Mail survey	Not reported, EU definition is not mentioned in questionnaire.	Mean number of rare disease patients per practice is 2.45 (SD 3.69) and median number is 1 (ranging from 0 to 21); 7/218 (3,2%) PHCP reported to have no rare disease patients
McClain *et al*. [9]	US, 2011	55 family practitioners of 592 (9.2%) with a membership of the the New Hampshire Academy of Family Physicians	Assess primary care pediatric providers' comfort with co-managing patients with rare conditions	Electronic survey concerning the care of children with rare and/or complex conditions	Rare conditions were defined as those occurring in less than 0.1% of children; complex conditions were defined as those involving ≥2 body systems and requiring on-going medical management.	No data
Miteva *et al*. [10]	Bulgaria, 2008	1,002 of a random sample of 2,042 GPs (49%)	Study the level of knowledge and general awareness on rare diseases among Bulgarian GPs	Telephone interviews	Not reported	4.2% of the GPs had seen ≥ 1 rare disease patients in last calendar year

Phillips [11]	US, year not reported	Selection of 100 patients with newly diagnosed rare disease from 4 family physicians in one practice	Describe the roles GPs play in the identification and management of patients with rare conditions in a typical practice	Office record review	"No objective definition of 'rare condition' is available…"	No data<br>Study goal "was not to estimate epidemiologic rates or to measure workload"
van de Laar *et al.*[12]	Nijmegen, Netherlands, 1986-2006	General practice patients included in the Continuous Morbidity Registration (CMR) Nijmegen (N=13,500)	Explore the prevalence of rare diseases and discuss methodological difficulties of studying rare disease in general practice	Extraction of diseases with a prevalence of < 0,5/1000 patient years from the CMR database	Results were compared with EU defined rare diseases	20 of 56 (36%) eligible codes referred to conditions that were listed as rare diseases in Orphanet
Van Nispen and Rijken [13]	Netherlands, baseline data from 1998 and additional data from 2002	206 rare disease patients from a nationwide representative sample of non-institutionalized chronic disease patients from 56 general practices	Describe health problems and quality of life experienced by people with uncommon chronic diseases, their GP treatment and their expectations	Mail and telephone interviews	Use of EU rare disease definition	72 rare diseases were described; 90% of rare disease patients had ≥1 GP contacts per year; Over 20% of rare disease patients experienced care needs not met by their GP and 16% needed more coordination of care by their GP

Table S2. List of 99 rare diseases reported for 121 active patients in the Belgian Network of SGP in 2015

Orphanet name for reported disease	N of active patients
46, XX ovotesticular disorder of sex development (DSD)	1
Addison disease	1
Aicardi-Goutières syndrome	1
Albright hereditary osteodystrophy	2
Alport syndrome	2
Amyotrophic lateral sclerosis (ALS)	3
Arachnoid cyst	1
Arthrogryposis multiplex congenita	1
Becker muscular dystrophy	1
Behçet disease	1
C1q esterase deficiency [2]	1
Central diabetes insipidus	1
Charcot-Marie-Tooth disease	2
Chondrodysplasia punctata - *peroxisomal disorder* [2]	1
Classic congenital adrenal hyperplasia due to 21-hydroxylase deficiency	1
Coats disease	1
Congenital Adrenal Hyperplasia	1
Congenital factor VII deficiency - *causing parahemophilia* [2]	1
Congenital hypophosphatemia [2]	1
Craniopharyngioma	1
Cutaneous lupus erythematosus	1
Cutaneous mastocytosis [1]	2
Cystinosis	1
Dermatomyositis	1
Disorder of bile acid synthesis	1
Duchenne	1
Dyskeratosis congenita (DC)	1
Ehlers-Danlos syndrome	2
Ehlers-Danlos syndrome type 2	1
Episodic ataxia type 5	1
Extraskeletal myxoid chondrosarcoma	1
Fabry disease	1
Familial adenomatous polyposis	1
Familial Mediterranean fever	2
Friedreich ataxia	2
Gastrointestinal stromal tumor (GIST)	1
Gaucher disease	1
Goodpasture syndrome	1
Hereditary leukodystrophy with axonal spheroids [2]	1
Familial spasmodic paraparesis [2]	1
Horton disease	1
Huntington disease	3
Hyper IgM syndrome [2]	1
Hypokalemic periodic paralysis	1

Hypophosphatasia	1
Idiopathic pulmonary fibrosis	1
Kallmann syndrome	1
Klippel-Trénaunay syndrome	1
Kugelberg-Welander disease	1
L-carnitine deficiency [1, 2]	1
Leber plus disease	1
Li-Fraumeni syndrome	1
Loeys-Dietz syndrome	1
Lupus [1,]	2
Mabry syndrome	1
MALToma	1
Maple syrup urine disease	2
Marfan syndrome	1
Medium chain acyl-CoA dehydrogenase (MCAD) deficiency	1
MELAS	2
Merosin negative congenital muscular dystrophy	1
Mitochondrial myopathy	1
Mowat-Wilson syndrome	1
Mucoviscidosis	2
Multiple acyl-CoA dehydrogenase deficiency (MADD)	1
Multiple endocrine neoplasia type 1 (MEN 1)	1
Multiple system atrophy	1
Muscular dystrophy	1
Myasthenia gravis	1
Neurofibromatosis [2]	3
Neurofibromatosis type 1	3
Neuronal ceroid lipofuscinosis	1
Osteogenesis imperfecta	1
Parapemphigus [2]	1
Paroxysmal nocturnal hemoglobinuria	1
Pleural mesothelioma	1
Poland syndrome	1
Postlingual non-syndromic genetic deafness	1
Prader-Willi syndrome	1
Primary biliary cirrhosis	1
Pseudoxanthoma elasticum	1
Pyoderma gangrenosum	1
Rare hereditary disease with peripheral neuropathy	1
Ring chromosome 20	1
Sarcoidosis	1
Scleroderma	2
Septo-optic dysplasia spectrum	1
Severe hemophilia A	1
Sickle cell anemia	2
Smith-Magenis syndrome	2
Sotos syndrome	1

Stargardt disease	1
Syringomyelia	1
Systemic mastocytosis	1
Trisomy 18 - *mosaic form* [2]	1
Turner syndrome	1
Waldenström macroglobulinemia	1
Wegener granulomatosis	1
Young-onset Parkinson disease	1

Notes: [1] The rare disease status could not be fully confirmed due to incomplete information provided by the GP. [2] Disease name not (yet) included in the ORPHA nomenclature.

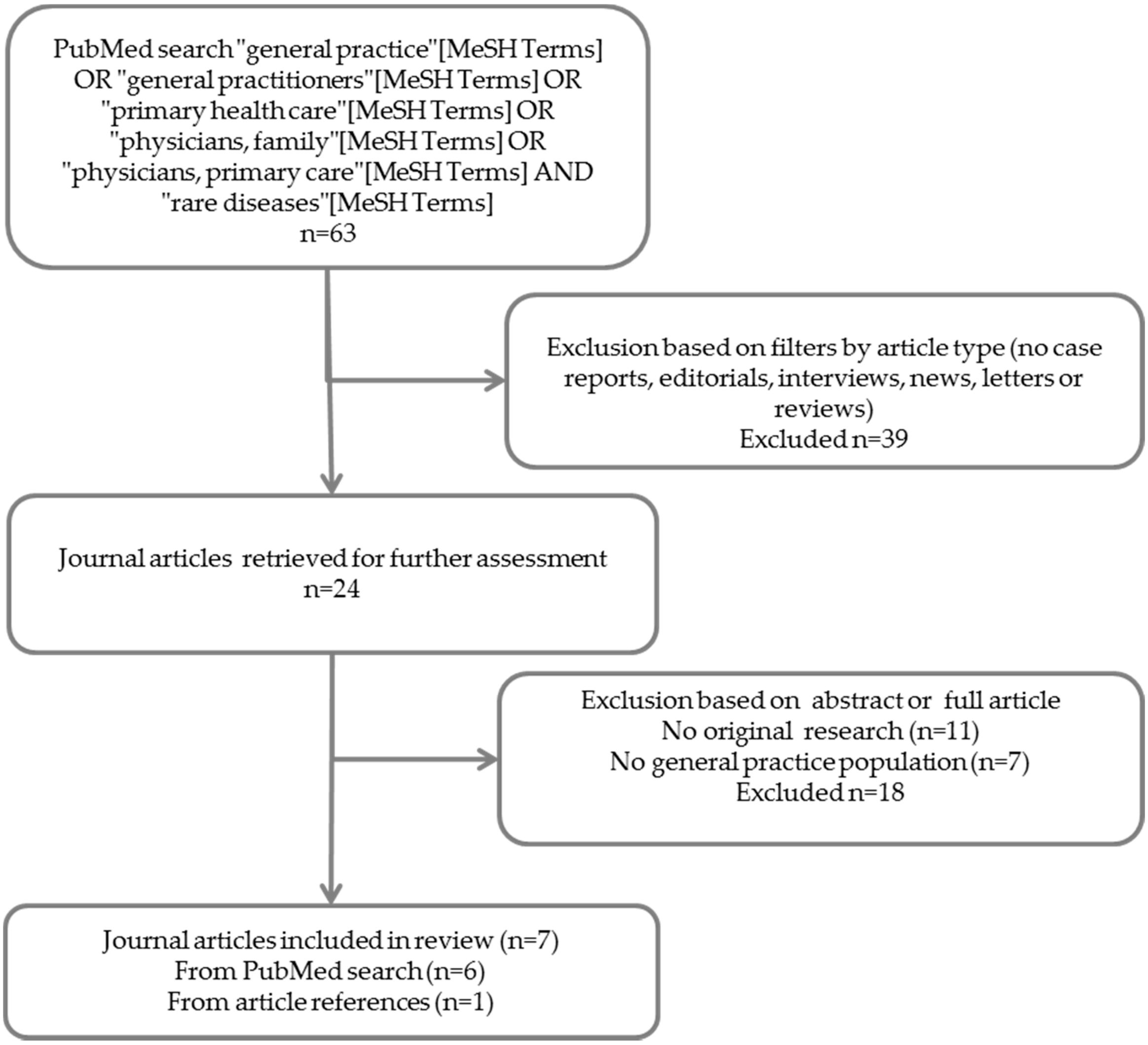

Figure S1. Flow chart of search for original studies on rare diseases in general practice
